# Late outcomes after acute pulmonary embolism: rationale and design of FOCUS, a prospective observational multicenter cohort study

**DOI:** 10.1007/s11239-016-1415-7

**Published:** 2016-08-30

**Authors:** Stavros V. Konstantinides, Stefano Barco, Stephan Rosenkranz, Mareike Lankeit, Matthias Held, Felix Gerhardt, Leonard Bruch, Ralf Ewert, Martin Faehling, Julia Freise, Hossein-Ardeschir Ghofrani, Ekkehard Grünig, Michael Halank, Nadine Heydenreich, Marius M. Hoeper, Hanno H. Leuchte, Eckhard Mayer, F. Joachim Meyer, Claus Neurohr, Christian Opitz, Antonio Pinto, Hans-Jürgen Seyfarth, Rolf Wachter, Bianca Zäpf, Heinrike Wilkens, Harald Binder, Philipp S. Wild

**Affiliations:** 1 Center for Thrombosis and Hemostasis, University Medical Center of the Johannes Gutenberg University, Langenbeckstrasse 1, Building 403, 55131 Mainz, Germany; 2 Department of Cardiology, Democritus University of Thrace, Xanthi, Greece; 3 Department of Cardiology, Heart Center at the University Hospital Cologne, and Cologne Cardiovascular Research Center, Cologne, Germany; 4 Abteilung für Innere Medizin, Missionsärztliche Klinik Würzburg, Würzburg, Germany; 5 Klinik für Innere Medizin und Kardiologie, Unfallkrankenhaus Berlin, Berlin, Germany; 6 Clinic for Internal Medicine, Greifswald University Hospital, Greifswald, Germany; 7 Klinik für Kardiologie, Angiologie und Pneumologie, Klinikum Esslingen, Esslingen am Neckar, Germany; 8 Klinik für Pneumologie, Medizinische Hochschule Hannover, Hanover, Germany; 9 Universitätsklinikum Gießen und Marburg, Marburg, Germany; 10 Thoraxklinik at Heidelberg University Hospital, Heidelberg, Germany; 11 Medizinische Klinik und Poliklinik I, Universitätsklinikum an der TU Dresden, Dresden, Germany; 12 Fachklinik für Innere Medizin, Krankenhaus Neuwittelsbach, Munich, Germany; 13 Department of Thoracic Surgery, Kerckhoff Heart and Lung Center, Bad Nauheim, Germany; 14 Lungenzentrum München, Klinik für Pneumologie und Pneumologische Onkologie, Klinikum Bogenhausen, Munich, Germany; 15 Medizinische Klinik und Poliklinik, LMU Klinikum der Universität München, Munich, Germany; 16 Klinik für Innere Medizin, DRK Kliniken Berlin Westend, Berlin, Germany; 17 Preventive Cardiology and Preventive Medicine, Center for Cardiology, University Medical Center of the Johannes Gutenberg University, Mainz, Germany; 18 German Center for Cardiovascular Research (DZHK), Partner Site RheinMain, Mainz, Germany; 19 Department of Pneumology, Universitätsklinikum Leipzig AöR, Leipzig, Germany; 20 Klinik für Kardiologie und Pneumologie, Universitätsmedizin Göttingen, Göttingen, Germany; 21 Saarland University Medical Center, Homburg, Germany; 22 Institute of Medical Biostatistics, Epidemiology and Informatics (IMBEI), University Medical Center of the Johannes Gutenberg University, Mainz, Germany

**Keywords:** Pulmonary embolism, Chronic thromboembolic pulmonary hypertension, Functional impairment, Quality of life, Cohort study, Follow-up

## Abstract

Acute pulmonary embolism (PE) is a frequent cause of death and serious disability. The risk of PE-associated mortality and morbidity extends far beyond the acute phase of the disease. In earlier follow-up studies, as many as 30 % of the patients died during a follow-up period of up to 3 years, and up to 50 % of patients continued to complain of dyspnea and/or poor physical performance 6 months to 3 years after the index event. The most feared ‘late sequela’ of PE is chronic thromboembolic pulmonary hypertension (CTEPH), the true incidence of which remains obscure due to the large margin of error in the rates reported by mostly small, single-center studies. Moreover, the functional and hemodynamic changes corresponding to early, possibly reversible stages of CTEPH, have not been systematically investigated. The ongoing Follow-Up after acute pulmonary embolism (FOCUS) study will prospectively enroll and systematically follow, over a 2-year period and with a standardized comprehensive program of clinical, echocardiographic, functional and laboratory testing, a large multicenter prospective cohort of 1000 unselected patients (all-comers) with acute symptomatic PE. FOCUS will possess adequate power to provide answers to relevant remaining questions regarding the patients’ long-term morbidity and mortality, and the temporal pattern of post-PE abnormalities. It will hopefully provide evidence for future guideline recommendations regarding the selection of patients for long-term follow-up after PE, the modalities which this follow-up should include, and the findings that should be interpreted as indicating progressive functional and hemodynamic post-PE impairment, or the development of CTEPH.

**Electronic supplementary material:**

The online version of this article (doi:10.1007/s11239-016-1415-7) contains supplementary material, which is available to authorized users.

## Unanswered questions after the acute phase of PE: the need for a large multicenter cohort study

Acute pulmonary embolism (PE) is a frequent cause of death and serious disability [1]. In an epidemiological model derived from six European countries, the estimated number of fatalities related to venous thromboembolism (VTE) amounted to 370,000, or 12 % of all deaths [2]. The risk of an adverse outcome during the acute phase varies widely depending on the clinical severity and the presence of right ventricular dysfunction at presentation [3], with early (30-day) mortality rates ranging from as low as 0.5 % in hemodynamically stable, ‘low-risk’ patients, to over 20 % in those presenting with cardiogenic shock [4]. Moreover, and importantly, the risk to die or develop persistent serious disability extends far beyond the acute phase of PE [5]. In earlier follow-up studies, as many as 25 % of the patients did not survive the first year after diagnosis, with the majority of deaths being related to underlying conditions such as cancer or chronic heart disease [6–8]. Consistently with these data, a cohort study of 866 patients who were retrospectively identified as having suffered acute PE (unprovoked in 308 patients; provoked in 558 patients), reported that 30 % of the study population died over a median follow-up period of 3.3 years [9]. Of 259 recorded deaths in that study, 110 were due to malignancy, 67 to recurrent PE, 30 to other cardiovascular disease, and the remaining 52 to other causes; in comparison, only 8.7 % of patients without PE died during the same study period [9]. Of note, there are no recent large multicenter cohort studies to provide contemporary, prospective data on long-term survival rates after PE.

Understanding and, as the following step, predicting the clinical course of a patient after acute PE is a far more complex task than answering the ‘dead or alive’ question. The available data from the follow-up of retrospectively identified patients, or from prospective studies of relatively small single-center cohorts (reviewed in [10]), suggest that more than 50 % of patients continue to complain of dyspnea and/or poor physical performance 6 months to 3 years after the index event, and up to 75 % perceive their own health status as being worse than before the acute PE episode. If confirmed in adequately powered follow-up studies, these numbers could be translated into a substantial clinical impact and socio-economic burden imposed by PE over the long term, far beyond that already acknowledged for the acute phase of the disease [11, 12]. At the far end of the severity spectrum of ‘late PE sequelae’ is a life-threatening disease termed chronic thromboembolic pulmonary hypertension (CTEPH). This progressive vasculopathy is thought to result from incomplete resolution of single or recurrent pulmonary emboli arising from sites of venous thrombosis [1, 13]. Although the diagnosis and management of CTEPH have made progress in recent years [14, 15], its true incidence and temporal pattern of development following an episode of acute PE remain obscure. The large margin of error in reported incidence rates of CTEPH (between 0.1 and 9.1 % within the first 2 years after a symptomatic PE event), is most likely due to referral bias, absence of early symptoms, and the difficulty in differentiating acute PE from an episode superimposed on pre-existing CTEPH [13, 16]. Moreover, and importantly, its ‘prodromi’, i.e. the clinical, functional and hemodynamic changes corresponding to early, possibly reversible stages of developing CTEPH, have not been systematically investigated.

The ongoing follow-up after acute pulmonary embolism (FOCUS) study will prospectively enroll and systematically follow, over a 2-year period and with a standardized comprehensive program of clinical, echocardiographic, functional and laboratory testing, a large multicenter prospective cohort of unselected patients (all-comers) hospitalized for acute symptomatic PE. FOCUS has been designed to possess adequate power which will enable it to provide answers to the above relevant remaining questions regarding the patients’ long-term morbidity and mortality after PE. It should thus be able to provide evidence for future guideline recommendations on which patients should be selected for long-term follow-up after PE, and possibly for ‘CTEPH screening’, and which modalities this follow-up should include.

## Study population and objectives of FOCUS

A total of 1000 consecutive patients with acute symptomatic, objectively diagnosed PE will be prospectively included in FOCUS on the basis of the eligibility criteria listed in Table 1. FOCUS explicitly aims to enroll ‘all-comers’ with PE, irrespective of the clinical severity, evidence of right ventricular dysfunction, or size or extent of pulmonary emboli. The primary objective is to determine the cumulative incidence of, (1) CTEPH, and (2) persisting or progressive functional and/or hemodynamic post-PE impairment (PPEI) over a 2-year follow-up period after an index episode of acute symptomatic PE. Secondary objectives are to assess, among others, overall and disease-specific long-term mortality, the incidence of major adverse cardiovascular events (such as acute myocardial infarction, stroke, or VTE recurrence), predictors as well as indicators of functional/hemodynamic impairment, fatal bleeding complications associated with long-term anticoagulant treatment for VTE, and the patients’ generic and disease-specific quality of life.

**Table 1:** Eligibility criteria

Inclusion criteria	Exclusion criteria
Objectively confirmed diagnosis of acute symptomatic PE by CTPA, V/Q lung scan, or invasive selective pulmonary angiography, according to established diagnostic criteria, with or without symptomatic DVT	Patients in whom the diagnosis of PE is an asymptomatic incidental finding during diagnostic work-up for another disease
Age ≥18 years	Patients with previously diagnosed CTEPH
Written informed consent obtained before enrollment	Previous enrollment in this study

*CTEPH* chronic thromboembolic pulmonary hypertension, *CTPA* computed tomographic pulmonary angiography, *DVT* deep vein thrombosis, *PE* pulmonary embolism, *V*/*Q* ventilation-perfusion

## Patient outcomes

The complete list of primary, secondary, and safety outcomes of FOCUS is provided in Table 2. Confirmed diagnosis of CTEPH during the 2-year follow-up period is the first primary outcome. However, as the occurrence of CTEPH is expected to be low and the absolute number of events small, even with this large patient population, PPEI has been defined as a co-primary outcome. This approach, which is explained in the statistical analysis section below, has its rationale in the assumption that persisting or progressive functional and/or hemodynamic impairment after acute PE is an early indicator of, and in statistical terms a ‘necessary condition’ for, the subsequent development of CTEPH. PPEI was defined by the FOCUS steering committee based on (and extrapolated from), (1) previously proposed prognostic criteria for pulmonary hypertension [17], (2) practical guides to cardiopulmonary exercise testing for evaluation of pulmonary hypertension and chronic thromboembolic disease [18, 19], and (3) the risk assessment of pulmonary (arterial) hypertension at follow-up visits as recommended in the 2009 [20] and recently updated [14] guidelines of the European Society of Cardiology and the European Respiratory Society. A detailed overview of the assessment and classification of individual indicators of PPEI ((a) and (b) parameters) is provided in Table 3.

All outcomes will be adjudicated by an independent Clinical Events Committee (CEC).

**Table 2:** Primary, secondary, and safety outcomes

Co-primary outcomes^a^	
(1) Confirmed diagnosis of CTEPH at any time during the 2-year follow-up	
(2) Post-PE impairment, defined by deterioration (compared to the findings at discharge, or to the previous follow-up visit) by at least one category, or persistence of the greatest severity category, in ≥1 of ‘a’ parameters plus deterioration by at least one category, or persistence of the greatest severity category, in ≥1 of ‘b’ parameters	
(a) Echocardiographic parameters of pulmonary hypertension and/or RV dysfunction^b^	(b) Clinical, functional and laboratory parameters of RV failure^b^
(a1) RV basal diameter (D1)	(b1) New appearance of symptoms or progression of existing symptoms
(a2) RA end-systolic area	(b2) Clinical evidence of RV failure
(a3) TAPSE	(b3) Syncope
(a4) LV eccentricity index	(b4) WHO functional class
(a5) Estimated RA pressure	(b5) Six-minute walking distance
(a6) Systolic TR jet velocity	(b6) BNP or NT-proBNP plasma levels
(a7) Pericardial effusion	(b7) Cardiopulmonary exercise testing

Secondary outcomes	
(1) Overall and disease-specific mortality during follow-up	
(2) Symptomatic recurrence of DVT or PE	
(3) Rehospitalization for reasons related to VTE, CTEPH, or complications of their treatment	
(4) New diagnosis of cancer	
(5) Acute myocardial infarction	
(6) Stroke	
(7) Functional limitation (peak O_2_ consumption and systolic blood pressure on cardiorespiratory exercise testing; six-minute walking distance and Borg dyspnea index)	
(8) Evidence of pulmonary hypertension/right ventricular dysfunction on echocardiography	
(9) Evidence of pulmonary vascular abnormalities on cardiopulmonary exercise testing^c^	
(10) Generic and disease-specific quality of life using the EQ-5D and the PEmb-QoL questionnaires	

Safety outcomes	
(1) Major bleeding during hospitalization for the index event, based on the ISTH definition [21]	
(2) Fatal bleeding at any time during follow-up	

*BNP* brain natriuretic peptide, *CTEPH* chronic thromboembolic pulmonary hypertension, *EQ-5D* Euro Quality of life five dimensions (questionnaire), *ISTH* International Society on Thrombosis and Haemostasis, *NT-proBNP* N-terminal pro-brain natriuretic peptide, *PE* pulmonary embolism, *PEmb-QoL* Pulmonary Embolism Quality of Life (questionnaire), *RA* right atrial, *RV* right ventricular, *TAPSE* tricuspid annular plane systolic excursion, *TR* tricuspid regurgitation, *VTE* venous thromboembolism, *WHO* World Health Organization

^a^See statistical analysis for details

^b^See Table 3 for severity classification of individual findings and parameters

^c^Indicated by at least one of the following: PETCO_2_ at AT (end-tidal partial carbon dioxide pressure at anaerobic threshold) <31.33 mmHg; P(a-ET)CO_2_ >5.18 mmHg; EQ O_2_ (oxygen ventilatory equivalent) >30.5; EQ CO_2_ (carbon dioxide ventilatory equivalent) >35.5; VE/VCO_2_ slope (ventilator efficiency for carbon dioxide) >37.5; P(A-a) O_2_ (alveolar–arterial oxygen gradient) >36.97 mm [22]

**Table 3:** FOCUS classification of the severity of post-PE abnormalities

Determinants of post-PE impairment	Normal or mild/low			Moderate			Severe/high	
(a) Echocardiographic parameters								
RV basal diameter (D1)	≤4.2 cm				>4.2 cm			
RA end-systolic area	≤18 cm^2^				>18 cm²			
TAPSE	≥1.6 cm				<1.6 cm			
Eccentricity index of LV	≤1.0				>1.0			
Estimated RA pressure	*0–5 mmHg* IVC diameter ≤2.1 cm and inspiratory collapse >50 %			*10 mmHg* IVC diameter ≤2.1 cm and collapse <50 %; or IVC diameter >2.1 cm and collapse >50 %			*15 mmHg* IVC diameter >2.1 cm and collapse <50 %	
Systolic TR jet velocity	<2.8 m/s			2.9–3.4 m/s			>3.4 m/s	
Pericardial effusion	No				Yes			
(b) Clinical, functional and laboratory parameters								
New appearance of symptoms or progression of existing symptoms	No				Yes			
Clinical evidence of RV failure	No				Yes			
Syncope	No				Yes			
WHO functional class	I or II				III or IV			
Six-minute walking distance	>500 m			300–500 m			<300 m	
BNP or NT-proBNP plasma levels	BNP <50 ng/L			BNP 50–300 ng/L			BNP >300 ng/L	
	NT-proBNP <300 ng/L			NT-proBNP 300–1400 ng/L			NT-proBNP >1400 ng/L	
Cardiopulmonary exercise testing (peak O_2_ uptake and systolic BP)^a^	>64.5 % and >120 mmHg	>64.5 % and ≤120 mmHg	46.3–64.5 % and >120 mmHg	46.3–64.5 % and ≤120 mmHg	34.1–46.3 % and >120 mmHg	34.1–46.3 % and ≤120 mmHg	<34.1 % and >120 mmHg	<34.1 % and ≤120 mmHg

*BNP* brain natriuretic peptide, *BP* blood pressure, *IVC* inferior vena cava, *LV* left ventricle, *NT-proBNP* N-terminal pro-brain natriuretic peptide, *RA* right atrial, *RV* right ventricular, *TAPSE* tricuspid annular plane systolic excursion, *TR* tricuspid regurgitation, *WHO* World Health Organization

^a^Peak O_2_ uptake as percentage of predefined value; systolic blood pressure measured at peak exercise

## Study design and flow

FOCUS is a prospective, multicenter, observational cohort study. The study protocol does not dictate any diagnostic or therapeutic interventions. On the other hand, the participating sites, which are high-volume centers with a long-standing experience in PE and pulmonary hypertension management, have harmonized their existing follow-up protocols and agreed that they will adhere to the follow-up schedule and workup presented in Table 4, which they consider as ‘best medical care’.

Detailed demographic and clinical data, diagnostic and therapeutic procedures, and outcome variables are recorded in an electronic case report form (eCRF). Regular follow-up visits are performed on discharge, and at 3, 12, and 24 months, as part of the best medical care standard at the participating centers.

**Table 4:** Data collection schedule

Variable	In-hospital		Follow-up		
	Enrollment	Discharge	3 months	12 months	24 months
Medical history	x				
Demographic data^a^	x				
Clinical examination^b^	x		x	x	x
Imaging (PE diagnosis)	x				
Echocardiography	x	x	x	x	x
Cardiopulmonary exercise testing			x	x	x
Laboratory diagnostic and safety tests^c^	x	x	x	x	x
Pharmacological treatment	x	x	x	x	x
Hemodynamic collapse	x	x	x	x	x
Survival status		x	x	x	x
Rehospitalization			x	x	x
Stroke		x	x	x	x
Symptomatic recurrent DVT/PE		x	x	x	x
Bleeding events		x	x	x	x
Functional status^d^			x	x	x
Diagnostic work-up for CTEPH^e^			x	x	x
Generic quality of life^f^			x	x	x
Disease-specific quality of life^g^			x	x	x

*CTEPH* chronic thromboembolic pulmonary hypertension, *DVT* deep vein thrombosis, *PE* pulmonary embolism

^a^Date of birth, gender, height, weight

^b^Presentation and symptomatology, vital signs; 12-lead ECG

^c^Serum creatinine, creatinine clearance [MDRD-estimate], TSH, O_2_-saturation [pulse oximetry], hematocrit, thrombocytes, leucocytes, aPTT, PT, INR, troponin T or I, NT-pro-BNP, BNP, CRP, d-dimer

^d^WHO functional class, Borg dyspnea index

^e^CT pulmonary angiography, V/Q scan; selective pulmonary angiography, right heart catheterization

^f^EQ-5D questionnaire [23]

^g^PEmb-QoL questionnaire [24]

## Biobanking substudy: the FOCUS BioSeq project

A multicenter biobanking substudy ‘Biochemical and Genetic Biomarkers in Sequelae of Acute Pulmonary Embolism Study (FOCUS BioSeq)’ is being conducted within the FOCUS cohort. Currently, a number of biomarkers have a place in PE management; most of them, including d-dimers, cardiac troponins, and natriuretic peptides, are used in the differential diagnosis or risk stratification of acute PE [1]. On the other hand, a systematic biochemical and genetic characterization of PE survivors over the long term is missing in the literature. Thus, the primary objective of the FOCUS BioSeq substudy is to identify and evaluate molecular and genetic markers for late sequelae of acute PE. Further objectives are to evaluate novel and established biomarkers for cost effectiveness and suitability in long-term management strategies, and as predictors of the response to pharmacotherapy.

**Fig. 1:**
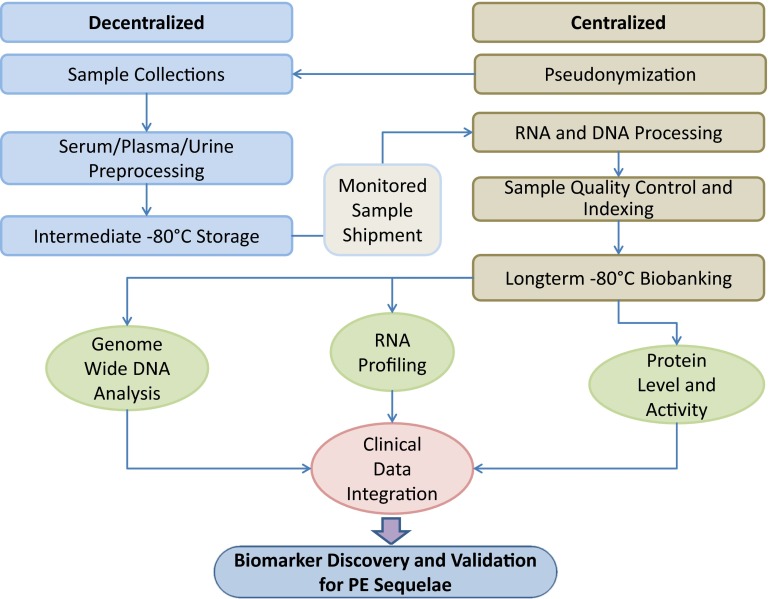
Biobanking workflow in the FOCUS BioSeq substudy

To achieve these goals, a decentralized and pseudonymized sample collection with centrifugal (standardized) preprocessing of plasma, serum and urine with short-term storage at −80 °C is being implemented in the participating study centers. For sample shipment to the central biobanking facility, pseudonyms and temperature control are maintained. On arrival, single sample aliquots are indexed for retrieval. Nucleic acids (DNA and RNA) are centrally isolated and quantity- and quality-controlled. Shipped, indexed and quality-controlled biomaterials are subsequently long-term stored in a centralized, 2-D barcoded and mirrored biobank at −80 °C. The following biomaterial specimens are collected in sufficient volumes for biomarker characterization and progression analysis in baseline and follow-up investigations: EDTA plasma, citrated plasma (3.2 %), serum, whole blood DNA and whole blood RNA (see Supplementary Table 1). Samples are collected and processed according to standard operating procedures (SOP). The FOCUS BioSeq biobanking procedures are illustrated in Fig. 1. Collection of biomaterial and optionally genetic examinations or exchange of biomaterial and data with collaboration partners are carried out after informed written consent has been provided by the study participant.

From the processed specimens, various biomarkers will be determined; a special focus will be put on biomarkers with known or expected relevance for the (pathophysiology of) circulation, hemostasis, inflammation, immunity, and kidney disease. High-throughput screening approaches will spotlight new potential biomarkers for sequelae of PE: Collected and purified DNA will be used for array-based genome-wide investigations aiming at discovering new genetic loci (further investigated by sequencing methods) which may predict the future clinical course of the disease or the response to drugs. Extracted RNA specimen like cellular mRNA, small non coding RNA or miRNA can be used for sequence probe based differential gene expression investigations. Epigenetic modifications on DNA samples or newly identified and known protein biomarkers at defined time points or in serial measurements from the follow-up assessment might be of prognostic relevance.

## Sample size calculation and statistical analysis

For sample size calculation, we assumed that the annual incidence rate of CTEPH in unselected patients who have suffered an episode of acute symptomatic PE (either provoked or unprovoked) is at least five times higher than the very low rate of 0.16 per 100 patients per year reported in one of the largest cohorts published to date [25], i.e. 0.8 per 100 patients per year. We further assumed that overall death rates and case-mix (high risk versus intermediate-risk versus low risk PE) will be similar to that previous study [25], and that there will be a ≤5 % loss-to-follow-up per year in addition to the administrative censoring and deaths. Following these assumptions, the cumulative incidence of CTEPH at 2 years is expected to amount to 1.3 %. In this case, our simulations indicate that a study population of 1000 patients will provide roughly 90 % power to reject the H_0_ hypothesis that the cumulative incidence of CTEPH at 2-year follow-up is 0.27 % (the cumulative incidence corresponding to the rate reported in [25]).

For a subgroup with an expected size of about 500 patients with unprovoked PE, i.e. an index PE event in the absence of reversible predisposing factors, an exploratory analysis will be performed. In this analysis, a power of more than 80 % can still be achieved to reject the H_0_ hypothesis that the cumulative incidence of CTEPH at 2-year follow-up is 0.27 %, if the hazard in the subgroup is at least six times higher than that previously reported for the unselected PE population [9, 25].

All enrolled patients will be included in the statistical analysis. Hierarchical testing will be used for the two co-primary outcomes, CTEPH and PPEI, assuming that all patients will present with PPEI before or at the time of the diagnosis of CTEPH. Therefore, we will first test whether the cumulative incidence of PPEI is significantly higher than 0.27 % at 2 years, and, if this is the case, whether the cumulative incidence for CTEPH also differs from the same threshold. The overall level of significance will be set to α = 0.05. Both tests will have a local power of approximately 90 %. Testing will be performed by checking whether the 95 % confidence interval of the 2-year Aalen-Johansen estimate of the cumulative incidence function (CIF) contains the value 0.27 %.

For the analysis of the primary outcome, it is assumed that CTEPH and PPEI can be considered independent of possible deaths during follow-up (e.g., mortality associated with cancer, myocardial infarction or stroke); if the cause of death is not independent from PPEI, the analysis for the primary outcome assumes that CTEPH or PPEI is always diagnosed before death. While these assumptions can reasonably be expected to hold true for PPEI, they might be less robust for CTEPH. Therefore, this latter point will be addressed by sensitivity analyses. The planned secondary and sensitivity analyses are listed in the Supplementary Material.

## Ethical aspects and data handling

The FOCUS study has been approved by the independent ethics committee at each participating site; it is being carried out in accordance with all local legal and regulatory requirements. All subjects must provide written informed consent to participate in the study, and a separate informed consent form is obtained for blood sampling and biodata banking. The study has been registered in the German Clinical Trials registry (http://www.germanctr.de; identifier: DRKS00005939).

As already mentioned, the study protocol does not dictate any diagnostic or therapeutic interventions; patients enrolled in FOCUS are treated according to current guidelines [1]. All findings, including clinical and laboratory data, are documented in individual medical records and in the eCRF. During entry of pseudonymized data, integrity checks help to minimize entry failures; any missing data or inconsistencies are reported back to the respective site and clarified by the responsible investigator. Regular monitoring is performed by personal visits from clinical monitors.

In case of withdrawal of a subject, the reason is documented and the patient is asked to consent to visit the clinic or a telephone interview in order to obtain as complete data as possible. Civil registers will be accessed or the patient´s family physician contacted if a personal contact is not possible.

## Innovative features and expected impact of FOCUS

Existing data suggest that incomplete thrombus resolution as well as persisting hemodynamic abnormalities and functional limitation over the long term occur quite frequently after acute PE. For example, between 30 and 52 % of the patients who survive the acute phase have been reported to have evidence of residual perfusion defects for at least 1 year after the acute event [26–29]; furthermore, the proportion of patients with some degree of persistent pulmonary hypertension after acute PE ranged between 40 and 69 % [30, 31]. However, these data are to be interpreted with caution, since the single-center cohort studies conducted thus far included rather small numbers of patients, the echocardiographic parameters used to assess the hemodynamic status post PE did not rigorously follow the standards proposed by scientific societies [32], and a correlation of ultrasound or thrombus imaging findings with the severity of the patients’ residual symptoms or the degree of functional limitation at follow-up could not be established [33]. These limitations unavoidably generate a high degree of uncertainty in estimating and predicting late outcomes after PE, more specifically the risk of developing CTEPH [5] as well as the entirety of persisting or progressive abnormalities belonging to the so-called ‘post-PE syndrome’ [10]. In this context, it also needs to be mentioned that some patients who have had PE may present with chronic symptomatic disease, which is morphologically indistinguishable from CTEPH but associated with normal pulmonary hemodynamics at rest (on echocardiography and right heart catheterization). Although these patients are also considered to have CTEPH and are managed accordingly, the pathophysiology of this ‘chronic thromboembolic pulmonary vascular disease’ remains obscure, and an exact definition and appropriate terminology is still lacking. In view of all these uncertainties, even the most recent guidelines are unable to provide clear recommendations on who, how often, and with what modalities should be followed after acute PE [1, 14].

In order to effectively address these clinically relevant issues, the FOCUS study is equipped, by its design, with the following innovative elements:

- a multicenter patient cohort with the participation of high-volume sites, geographically distributed all over Germany;
- participating sites which possess recognized experience in the management of diseases of the pulmonary circulation and the right ventricle, and all fulfill high standards of patient care;
- although FOCUS is not an interventional study and thus does not, per se, dictate diagnostic or therapeutic measures, the participating sites have standardized and harmonized their existing follow-up programs to reflect the ‘best current medical care’ of patients having suffered acute PE;
- prospective enrollment of unselected ‘all-comers’ with acute symptomatic PE and prospective collection of data during multiple visits at predefined intervals;
- a state of the art biobanking program;
- a relatively short recruitment period of approximately 2 years in order to exclude the ‘confounder’ of major changes in the diagnostic and therapeutic approach to acute PE or in the follow-up phase during the study;
- contemporary management of acute PE and CTEPH, reflecting the increasing role of the novel, direct oral anticoagulants, and implementing up-to-date algorithms for diagnostic workup of suspected CTEPH and patient management upon confirmation of the disease;
- adequate sample size calculated to permit a reliable assessment of CTEPH rates after PE; even more important, prospective definition, as a co-primary outcome, of ‘post-PE impairment’ based on combinations of clinical, functional, hemodynamic, and imaging abnormalities which may persist or develop after the index episode; thus, the patients’ clinical course after acute PE is no longer reduced to a ‘yes or no’ question for CTEPH, but will be considered as a continuum of late post-PE complications;
- sufficient power to determine clinical, functional, echocardiographic and laboratory determinants of CTEPH and/or PPEI.

As of August 2016, a total of 602 patients have been included at 19 active sites across Germany with a dropout rate of <5 %. Enrollment of the last patient is expected by the fourth quarter of 2017. The results of FOCUS and FOCUS BioSeq are expected to have a significant impact on the long-term management of patients after acute PE.

## Electronic supplementary material

Below is the link to the electronic supplementary material.

Supplementary material 1 (DOCX 17 KB)
