# Supplementary material for: Late outcomes after acute pulmonary embolism: rationale and design of FOCUS, a prospective observational multicenter cohort study
Source: J Thromb Thrombolysis. 2016 Aug 30;42(4):600–9. doi: 10.1007/s11239-016-1415-7 (PMC5040729; doi:10.1007/s11239-016-1415-7)
Supplement: Supplementary file 1 — Supplementary material 1 (DOCX 17 KB) [file 11239_2016_1415_MOESM1_ESM.docx]

*Supplementary material*

**Late Outcomes After Acute Pulmonary Embolism: Rationale and Design of FOCUS, a Prospective Observational Multicenter Cohort Study**

Stavros V. Konstantinides, MD, PhD, Stefano Barco, MD, PhD, Stephan Rosenkranz, MD, PhD, Mareike Lankeit, MD, Matthias Held, MD, Felix Gerhardt, MD, Leonard Bruch, MD, Ralf Ewert, MD, Martin Faehling, MD, Julia Freise, MD, Hossein-Ardeschir Ghofrani, MD, Ekkehard Grünig, MD, Michael Halank, MD, Nadine Heydenreich, PhD, Marius M. Hoeper, MD, Hanno H. Leuchte, MD, Eckhard Mayer, MD, F. Joachim Meyer, MD, Claus Neurohr, MD, Christian Opitz, MD, Antonio Pinto, PhD, Hans-Jürgen Seyfarth, MD, Rolf Wachter, MD, Bianca Zäpf, MSc, Heinrike Wilkens, MD, Harald, Binder, PhD, and Philipp S. Wild, MD, MSc

**Corresponding author**

Stavros V. Konstantinides, MD, PhD

Center for Thrombosis and Hemostasis, University Medical Center Mainz

Langenbeckstrasse 1, Building 403, 55131 Mainz, Germany

E-mail: [stavros.konstantinides@unimedizin-mainz.de](mailto:stavros.konstantinides@unimedizin-mainz.de)

**Supplementary Table 1.** Collection of biomaterial specimens for biobanking in FOCUS BioSeq.

| **Biomaterial**  **Specimen** | **Acute PE** | **Follow Up** | | | **Collection**  **Tube** |
| --- | --- | --- | --- | --- | --- |
|  | **In Hospital** | **3 Month** | **12 Month** | **24 Month** |  |
| EDTA Plasma | 9 ml | 9 ml | 9 ml | 9 ml | Sarstedt Monovette, 9 ml, K3 |
| Citrate Plasma 3.2% | 9 ml/18 ml* | 9 ml/18 ml* | 9 ml/18 ml* | 9 ml/18 ml* | Sarstedt Monovette 10 ml, 9NC |
| Serum | 7.5 ml | 7.5 ml | 7.5 ml | 7.5 ml | Sarstedt Monovette 7,5 ml, Z |
| Whole Blood RNA | 2.5 ml | 2.5 ml | - | - | PAXgene RNA Blood |
| Whole Blood DNA | 8.5 ml | 8.5 ml | - | - | PAXgene DNA Blood |
| Citrate Plasma 3.8% | -/3.8 ml* | -/3.8 ml* | -/3.8 ml* | -/3.8 ml* | Sarstedt Monovette 3,8 ml, 9NC (PFA) |
| Urine | 5 ml | 5 ml | 5 ml | 5 ml | Boettger  Urine Beaker 125 ml |

Blood volumes marked with asterisk (*) are only collected at the central biobanking site.

### Statistical methods: secondary and sensitivity analyses

1. All analyses will be performed also without including patients who experience CTEPH but no post-PE impairment (PPEI) as a sensitivity analysis.
2. A competing risk analysis for sensitivity analysis, whether the assumption of no association between death and undiagnosed CTEPH or PPEI can be assumed, will be performed.
3. More general multi-state-models for modeling the progression of the disease and the influence of different states of the disease and primary diseases on the risk of PPEI, to get a better understanding for whom and when a close monitoring is appropriate, will be provided.
4. Aalen-Johansen estimators for cumulative incidence of CTEPH and incidence of PPEI with corresponding 95% confidence intervals will be calculated.
5. To address potential effects of the degree of center specialization in PPEI and CTEPH incidence, these incidences will also be calculated after re-weighting the centers according to their case-mix. This allows estimation of incidences standardized to a health services setting with a different kind of specialization distribution, i.e. to account for a potential overrepresentation of highly specialized centers in the study.
6. For the incidence of PPEI in different types of subgroups, such as provoked vs. unprovoked or cancer patients vs other patients Aalen-Johansen estimators plus 95% confidence intervals will be calculated. If risk factors occur during the follow-up, they will be taken into account using time-dependent covariates.
7. Sensitivity analysis for the definition of PPEI will be performed.
